# Chitinase Is Involved in the Fruiting Body Development of Medicinal Fungus *Cordyceps militaris*

**DOI:** 10.3390/life13030764

**Published:** 2023-03-12

**Authors:** Zi-Juan Zhang, Yuan-Yuan Yin, Yao Cui, Yue-Xuan Zhang, Bi-Yang Liu, You-Chu Ma, Yong-Nan Liu, Gao-Qiang Liu

**Affiliations:** 1 Hunan Provincial Key Laboratory of Forestry Biotechnology, Central South University of Forestry & Technology, Changsha 410004, China; 2 International Cooperation Base of Science and Technology Innovation on Forest Resource Biotechnology of Hunan Province, Central South University of Forestry & Technology, Changsha 410004, China; 3 Microbial Variety Creation Center, Yuelushan Laboratory of Seed Industry, Changsha 410004, China

**Keywords:** *Cordyceps militaris*, chitin degrading enzyme, cell wall, mushroom formation

## Abstract

*Cordyceps militaris* is a famous traditional edible and medicinal fungus in Asia, and its fruiting body has rich medicinal value. The molecular mechanism of fruiting body development is still not well understood in *C. militaris*. In this study, phylogenetically analysis and protein domains prediction of the 14 putative chitinases were performed. The transcription level and enzyme activity of chitinase were significant increased during fruiting body development of *C. militaris.* Then, two chitinase genes (*Chi1* and *Chi4*) were selected to construct gene silencing strain by RNA interference. When *Chi1* and *Chi4* genes were knockdown, the differentiation of the primordium was blocked, and the number of fruiting body was significantly decreased approximately by 50% compared to wild-type (WT) strain. The length of the single mature fruiting body was shortened by 27% and 38% in *Chi1-* and *Chi4-*silenced strains, respectively. In addition, the chitin content and cell wall thickness were significantly increased in *Chi1-* and *Chi4-*silenced strains. These results provide new insights into the biological functions of chitinase in fruiting body development of *C. militaris.*

## 1. Introduction

*Cordyceps militaris* is a well-known traditional edible and medicinal mushroom in Asia. Its fruiting body has the functions of anti-tumor [1], protecting the liver and kidney [2], resisting influenza virus [3] and inducing apoptosis of cancer cells [4]. Methods for large-scale industrial production of *C. militaris* fruiting body are gradually established in artificial media, such as rice [5] or with insects, such as silkworm Bombyx mori pupae [6]. However, *C. militaris* have low yield under industrial conditions partially due to little is known about the how fruiting body development is regulated. To this end, elucidating the regulation mechanism of fruiting body development has become a hot research topic in *C. militaris*.

In order to identify the genes involved in fruiting body development in *C. militaris*, screening of a random T-DNA insertion mutant library showed that ATP-dependent helicase, cytochrome oxidase subunit I, ubiquitin-like activating enzyme and serine/threonine phosphatase were identified to be involved in fruiting body production [7]. The vegetative growth of a *CmSnf1* deletion mutant (Δ*CmSnf1*) was reduced by 42.2% with arabinose as a sole carbon source in *C. militaris*, and *CmSnf1* was necessary for mycelium to penetrate the insect cuticle to form the fruiting body on silkworm pupae, consistent with the down-regulation of chitinase- and protease-encoding genes in Δ*CmSnf1* [8]. The Δ*Cmcrf1* and Δ*Cmfhp* mutant cultured on rice medium did not produce fruiting bodies and did not have an impact on the mycelia growth of *C. militaris* [9,10]. In addition, light is an essential factor for pigment formation and fruiting body development in *C. militaris* [11]. However, the molecular mechanism of fruiting body formation of *C. militaris*.is still not well understood.

Chitin, a structural endogenous carbohydrate, is a major component of fungal cell walls. In several groups of fungi, chitin replaces cellulose as the structural polysaccharide [12]. Fungal chitinases are known to belong only to the glycoside hydrolase family 18 (GH18) family, whose function is to degrade endogenous chitin in chitin-containing organisms [13,14]. Chitinase-mediated degradation of chitin plays an important role in fungi organismal development, phytopathogenicity, and cell wall stress. In pathogenic fungi *Beauveria bassiana*, knock out two chitinase genes *Blys5* or *Blys2*, containing conserved lysin motif (LysM) characterized as a virulence factor, results in significantly weakened of infection toxicity [15]. In *Lentinula edodes*, the genes encoding chitinolytic enzymes and chitinase activities were increased after mushroom harvest and transcriptome analysis revealed that many cell wall-related enzymes were upregulated after fruiting body harvest, such as β-1,3-1,6-glucan-degrading enzymes, putative chitinases in GH18, GH20, and GH75 [16]. In other hands, down-regulation of cell wall chitinase and β-1,3 glucanase by treated with a solution of CaCl_2_ and citric acid delayed the fruiting body softening of *Agaricus bisporus* [17]. Particularly, a reference atlas of mushroom formation based on developmental transcriptome data of six species and comparisons of >200 whole genomes were constructed, revealed nearly 300 conserved gene families and >70 functional groups contained developmentally regulated genes (including chitinase), targeted protein degradation, signal transduction, etc. [18]. While, the role of chitinase in fruiting body formation of *C. militaris* and other mushrooms remains unclear.

To this end, in this study, the GH18 chitinase family was characterized by bioinformatics, physiology and genetic analysis in *C. militaris*. The transcription level of 14 putative chitinase genes, and chitinase activity were evaluated in six different developmental stages of *C. militaris*. Then, two chitinase genes (*Chi1* and *Chi4*) were selected to construct gene silencing strain by RNA interference (RNAi). The fruiting body development, chitin content, and cell wall thickness were examined in the *Chi1*- and *Chi4*-silenced strains. Our results showed that chitinase-mediated chitin degradation is essential for fruiting body development in *C. militaris*.

## 2. Materials and Methods

### 2.1. Strains and Culture Conditions

The *C. militaris* strain (ACCC53264, Chinese agricultural culture collection) used in this study was cultured on a potato glucose agar (PDA) medium containing 15 g/L agar at 28 °C. IM medium (IM contains 228 g/L K_2_HPO_4_·3H_2_O, 68 g/L KH_2_PO_4_, 14.6 g/L NaCl, 49 g/L MgSO_4_·7H_2_O, 10 g/L CaCl_2_·2H_2_O, 53 g/L (NH_4_)_2_SO_4_, 5.5 g/L FeSO_4_·7H_2_O) was used to co-cultivation Agrobacterium (EHA105) and chitinase-silenced strains of *C. militaris*. M-100 medium was used to culture chitinase-silenced strains of *C. militaris* (M-100 contains 62.5‰ M-100 salt solution, 10‰ glucose, 3‰ KNO_3_; M-100 salt solution contains 16‰ KH_2_PO_4_, 4‰ Na_2_SO_4_, 8‰ KCl, 2‰ MgSO_4_·7H_2_O, 1‰ CaCl_2_, 8‰ M-100 trace element solution; M-100 trace element solution contains 0.06‰ H_3_BO_3_, 0.14‰ MnCl_2_·4H_2_O, 0.4‰ ZnCl_2_, 0.04‰ Na_2_MoO_4_·2H_2_O, 0.1‰ FeCl_3_·6H_2_O, 0.4‰ CuSO_4_·5H_2_O). Escherichia coli strain DH5α was grown in Luria-Bertani (LB) medium containing 100 μg/mL ampicillin at 37 °C was used for plasmid amplification. In vitro fruiting body production of *C. militaris* was performed with an artificial medium containing 20 g rice, 0.5 g powder of silkworm pupae and 25 mL nutrient solution (glucose 20 g, KH_2_PO_4_ 2 g, MgSO_4_ 1 g, ammonium citrate 1 g, peptone 5 g, vitamin B1 20 mg, and 1000 mL distilled water), according to the previous method [7]. After cultured for 25 days at 25 °C in dark, the *C. militaris* mycelia were transferred to 20 °C, lighting for 12 h and ventilation, to induce the color change of hyphae promoted the formation of cones and fruiting body. Take samples from five different development stages, white vegetative mycelium, yellow vegetative mycelium, primordium, young fruiting body, and mature fruiting body on the 25th, 35th, 50th, 60th and 70th day.

### 2.2. Homology Comparison and Bioinformatics Analysis

The predicted chitinase proteins were subjected to BLASTp against the non-redundant (nr) database at the National Centre for Biotechnology Information (NCBI) using an E value cutoff of 10^−5^. The draft genome of C. militaris was encoded at NCBI GenBank under the accession number AEVU00000000 [19]. Download the base sequence of chitinase from the NCBI website, carry out multiple sequence alignment in KEGG, the evolutionary history was inferred by the neighbor-joining method. The bootstrap consensus tree, inferred from 1000 replicates, represents the evolutionary history. Phylogenetic analyses were conducted with MEGA 7 software. The domain architecture of chitinases were annotated using the Pfam v27.0 databases (http://pfam.xfam.org/search (accessed on 1 May 2020), E value < 1.0).

### 2.3. cDNA Preparation and qRT-PCR

RNA isolation and cDNA synthesis were performed as previously described method [20]. Briefly, 0.2 g mycelia were collected and subsequently were disrupted under liquid nitrogen conditions. The RNA Isolation Kit (TaKaRa, Beijing, China) was used to extract total RNA. Next, 2 μg RNA was treated with DNase I which was used to avoid genomic DNA contamination, and cDNA was synthesized using 20 μL reverse transcription reaction system following the manufacturer’s instruction. qRT-PCR experiments were performed using a previously described method [21]. Each treatment included three biological replicates and three technical repetitions. The relative mRNA levels of the target genes were calculated using the Livak calculation method (2^−ΔΔCt^) by normalizing them to the expression of the screened reference gene [22]. All the PCR primers were designed using the Primer Premier 5 software. The gene specific primers used are listed in Table S1 (Supplementary Materials).

### 2.4. Chitinase Activity Assay Method

The *C. militaris* mycelium is washed three times with PBS, and ground into powder with liquid nitrogen for protein extraction. A chitinase activity assay kit (catalog no. CS0980; Sigma-Aldrich, St. Louis, MO, USA) was used with 4-nitrophenyl N-acetyl-β-D-glucosaminide (a substrate suitable for exochitinase activity detection), as instructed by the manufacturer [23]. Unit definition (U): one unit will release 1.0 mmole of p-nitrophenol from the appropriate substrate per minute at pH 4.8 at 37 °C. Chitinase activity (U/mg) = [(A_405_sample − A_405_blank) × 0.05 × 0.3 × DF]/[A_405_standard × time × Venz × Total protein]. A_405_sample: absorbance of the sample at 405 nm; A_405_blank: absorbance of the blank at 405 nm; 0.05: mmole/mL of p-nitrophenol in the standard solution; 0.3: final volume of the 96 well plate reaction after addition of the stop solution (mL); DF: dilution factor; A_405_standard: absorbance of the standard solution at 405 nm; time: minutes; Venz: volume of the sample (mL). Asterisks indicate significant differences (* *p* < 0.05, ** *p* < 0.01) compared to the control according to two-way analysis of variance (ANOVA) using GraphPad Prism. The values were expressed as the mean ± SD, and *p* < 0.05 was defined as statistically significant.

### 2.5. Determination of Chitin Content

The determination of chitin content is as previously described [24]. Briefly, the ground *C. militaris* mycelium powder was collected and resuspended in 3 mol/L KOH in a water bath at 80 °C for 90 min, and then pre-cooled 75% ethanol was added in advance and then the powder was placed in an ice bath for 15 min on an ice box. Then 545 diatomite (mass to volume ratio 13.3%) was added and mixed, and the precipitation was collected by centrifugation. The precipitate was washed with pre-cooled 40% ethanol and then washed twice with pre-cooled ddH_2_O. The precipitate was resuspended in water, 5% KHSO_4_ and 5% (*w*/*v*) NaNO_2._ The supernatant was centrifuged and mixed with ddH_2_O and 12.5% (*w*/*v*) (NH_4_)_2_SO_4_. Next, 5 mg/mL 3-methyl-2-benzothiazolinone hydrazone hydrochloride was added, boiled for 5 min, 0.83% (*w*/*v*) FeCl_3_ was added, and the reaction was carried out at room temperature for 25 min. The absorbance value was measured by microplate reader at 650 nm. Standard curves were drawn using glucosamine as a standard.

### 2.6. Construction of RNAi Strains

Construction of RNA interference cassette targeting chitinase using fungal RNAi vector pCAMBIA1300. Inhibition of chitinase expression by glyceraldehyde-3-phosphate dehydrogenase promoter and 35S promoter. The chitinase gene was amplified by PCR using *C. militaris* cDNA as the template and the primers listed in Table S1, and the silent fragment primers of the conserved regions of *Chi1* and *Chi4* were designed. The obtained PCR product was added to the agarose gel with dye solution. A gel imager was used for testing and the DNA gel recovery kit was used to recover it. T-A cloning vector was connected for 12 h, and inhaled the vector connected to the silent DNA segment for *Escherichia coli* DH5α, stranded for 25–30 min, heat shocked for 60 s, and the sterile LB medium without antibiotics was added to mixed evenly and cultured for 1 h. The supernatant was then centrifuged for 30 s and discarded. After the suspended cells were suspended, they were coated on LB solid medium containing kanamycin (50 mg/mL), and inverted at 37 °C to culture. The plasmid of the silent vector was extracted and the plasmid DNA of *Chi1* and *Chi4* was digested by BamHI and SalI to obtain the double digested products, then they were contected with T4 DNA ligase and transform the enzyme linked products into *E. coli* DH5α. PCR detection was performed in PCAMBIA1300 plasmid. Inoculated EHA105 Agrobacterium in YEB, and incubated in a shaking table for 12–16 h. Inoculated recovered *Agrobacterium tumefaciens* into YEB, and continued to culture until OD_600_ is 0.4–0.5. *Agrobacterium* EHA105 was inoculated in YEB to prepare *Agrobacterium* competent cells, the successfully constructed pCAMBIA1300 plasmid (RNAi: pGPD-CmChitinase-p35S) was transferred into *Agrobacterium* EHA105. Then prepared pupae grassland protoplasts, and 100 μL *Agrobacterium* and 100 μL protoplasm whose concentration was up to standard were fully mixed, then coated them on IM solid medium covered with cellophane, and co-cultured in dark for 2 days. The plasmid RNAi: pGPD-CmChitinase-p35S was transformed into *C. militaris* by *Agrobacterium tumefaciens*-mediated transformation (ATMT). After the co-culture, dozens of transformants were randomly selected after growth on M-100 medium containing 50 mg/mL carbenicillin and 100 mg/mL hygromycin B. qRT-PCR analyses were performed to determine the silencing efficiency of the transformants. Gene expression was evaluated by calculating the difference between the threshold cycle (CT) value of the gene analyzed and the CT value of the housekeeping gene tubulin (TUB). qRT-PCR calculations analyzing the relative gene expression levels were performed according to the 2^−ΔΔCT^ method with paired primes listed in Table S1.

### 2.7. Transmission Electron Microscopy (TEM)

The detection of cell wall thickness by transmission electron microscopy is a reference to previously reported methods with some modifications [25]. Mycelia were collected into phosphate buffer pH 7.4 0.1 M containing 2.5% glutaraldehyde and fixed for 24 h. They were subsequently washed with 0.1 M PBS and fixed for 1 h on ice in 1% osmium tetroxide, and then completely dehydrated with 70%, 80%, 90%, and 100% ethanol, cleared in propylene oxide, and macerated overnight with a 1:1 ratio of propylene oxide: Spurr’s resin. After 2–3 h maceration in pure resin (Spurr’s resin-Eponate 12, 1:1), it was cured in 60% oven for 48 h. The ultrathin sections of 80 nm were collected on the grid and stained successively with 1% tannic acid (filtered aqueous solution), 2% uranyl acetate (filtered aqueous solution) and lead citrate. The ultrathin sections were observed under a Hitachi 7650 transmission electron microscope at 80 kV. Photographs were taken with a built-in amt8000 × 8000 charge-coupled device camera.

## 3. Results

### 3.1. Character of Chitinase in Fruiting Body Development of C. militaris

**Figure 1:**
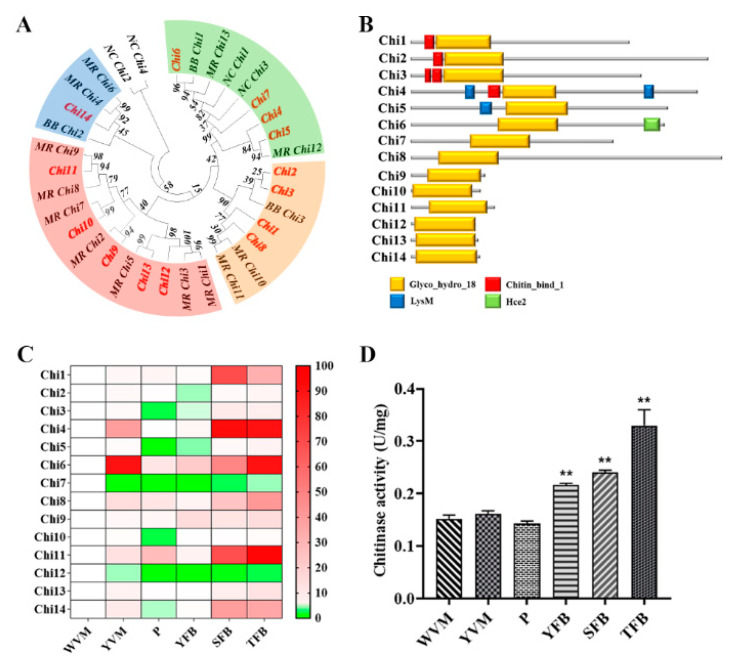
Character of chitinase in fruiting body development of *C. militaris.* (**A**) A dendrogram showing the relationships between the *Chi1–14* gene and the related sequences retrieved from the *M. robertsii*, *B. bassiana* and *N. crassa* chitinase. The accession numbers of the *MR_Chi1–13* are EXU94655.1, EXV00011.1, XP_007819649.1, EXU97041.1, XP_011411561.1, XP_007826104.1, XP_007820987.1, EXU96979.1, XP_007818874.1, EXU94868.1, EXU95642.1, XP_007817839.2, and EXV01258.1. *NC_Chi1–5*: XP_965031.2, HBG51784.1, XP_961930.1, P36981.2, and KHE80352.1. *BB_Chi1–3*: AIT18887.1, QIZ03092.1, and AIT18874.1. *Chi1–14:* XP_006673432.1, XP_006665541.1, XP_006668866.1, XP_006670325.1, XP_006670026.1, XP_006674319.1, XP_006674376.1, XP_006674041.1, XP_006673374.1, XP_006665708.1, XP_006672675.1, XP_006668493.1, XP_006671275.1, and XP_006674325.1. (**B**) The conserved domain prediction of 14 putative chitinase in *C. militaris.* Glycosyl hydrolases family 18 domain (Glyco_hydro_18, GH18, marked in yellow); Type 1 chitin binding domain (Chitin_bind_1, marked in red); Lysin motif domain (LysM, marked in blue); Homolog of *Cladopsorium fulvum* Ecp2 effector domain (Hce2, marked in green). (**C**) The expression of 14 chitinase genes at 6 different developmental stages (white vegetative mycelium, WVM; yellow vegetative mycelium, YVM; primordium, P; young fruiting body, YFB; stipe of mature fruiting body, SFB; tip of mature fruiting body, TFB). Chitinase genes were detected using qRT-PCR and were presented as a microarray heatmap. The relative expression is shown as a mean value difference from 0.0 to 100.0 in green to red. The expression level of each chitinase genes in WVM was arbitrarily set to 1. (**D**) Chitinase activity during 6 stages of fruiting body development. The mean and standard error were determined using data from three independent replicates (*n* = 3, ** *p* < 0.01 by two-way ANOVA).

To investigate the role of chitinase in *C. militaris*, the sequences of the chitinase gene of *Metarhizium robertsii* (MR, GenBank accession number: XP_011411561.1), *Beauveria bassiana* (BB, AIT18887.1) and *Neurospora crassa* (NC, XP_961930.1) were used as input in the BLAST search of the *C. militaris* genome database. Fourteen putative chitinase genes (named as *Chi1–14*) were obtained. Then, 13 chitinases from *M. robertsii*, 5 chitinases from *N. crassa* and 3 chitinases from *B. bassiana* were used for phylogenetic analysis with *Chi1–14* (Figure 1A). An evolutionary relationship analysis indicated that the protein sequences encoded by *Chi1–3* and *Chi8* were observed to cluster with *MR_Chi10–12* and *BB_Chi3*. The protein sequences encoded by *Chi4–7* were clustered with the *MR_Chi12*, *MR_Chi13*, *NC_Chi1*, *NC_Chi3* and *BB_Chi1*. The protein sequences encoded by *Chi9–13* were clustered with the*MR_Chi1–3*, *5* and *MR_Chi7–9*. The protein sequences encoded by *Chi14* was clustered with the *MR_Chi4*, *MR_Chi6* and *BB_Chi2*. *NC_Chi2* and *NC_Chi4* were clustered together separately. Conserved domain analysis showed that *Chi1–14* proteins contained glycosyl hydrolases family 18 domains. *Chi1–4* contained type 1 chitin binding domain. The *Chi4* and *Chi5* contained lysin motif domain. Only *Chi6* contained Hce2 domain. All the above domains belong to the typical chitinase domain found in other fungi [26]. These results indicated that the *Chi1–14* was a putative chitinase of *C. militaris*.

Next, real-time quantitative PCR was used to detect the expression of 14 chitinase genes in six fruiting body development stages of *C. militaris*. As shown in Figure 1B, the gene expression levels of *Chi1*, *Chi4*, *Chi6*, and *Chi11* were significantly increased during fruit body development, while the gene expression levels of *Chi7* and *Chi12* were significantly decreased during fruit body development. Furthermore, the chitinase activity in six fruiting body development stages were assayed. As shown in Figure 1C, the chitinase activity was significantly increased during the fruiting body development of *C. militaris*. Compared to white vegetative mycelium (WVM), the chitinase activity of mature fruiting body (MFB) was significantly increased approximately by 2.1-fold. These results hint that chitinase play a role in fruiting body development in *C. militaris*. Then, the significantly upregulated chitinase genes *Chi1* and *Chi4*, which containing GH18 and chitin binding domains, were selected for follow-up study.

### 3.2. Disruption of Chitinase in C. militaris

**Figure 2:**
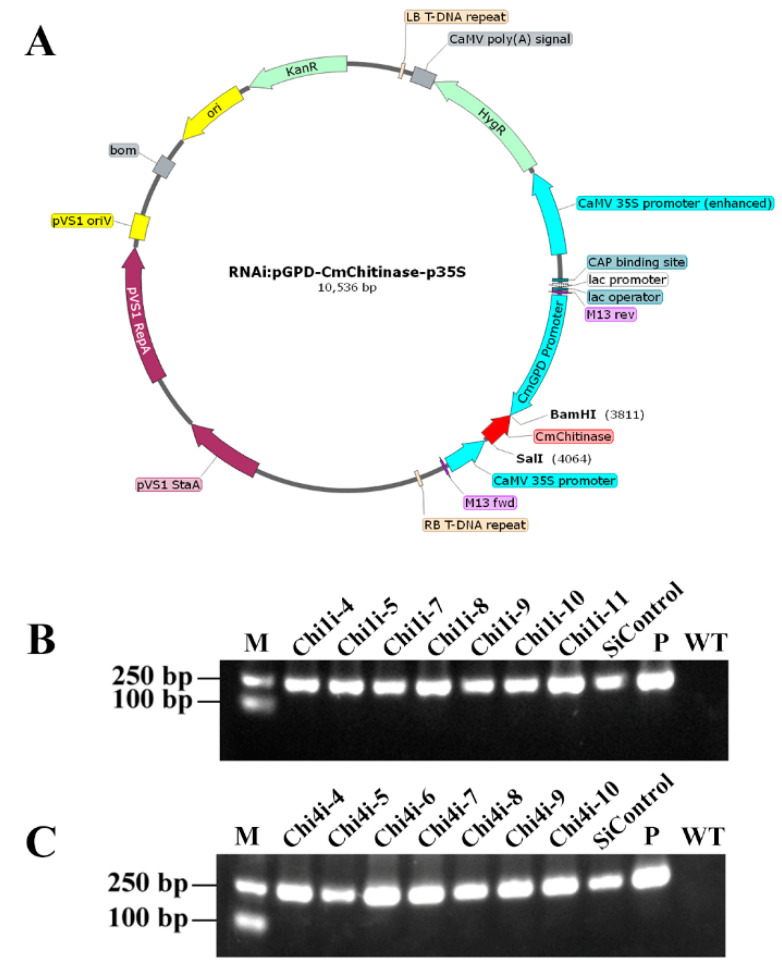
Construction of chitinase gene silencing strain in *C. militaris*. (**A**) Construction of *chi*tinase silencing plasmid. The ~200 bp gene sequence of *Chi1* and *Chi4* was encoded by the dual promoters of CaMV 35S promoter and CmGPD promoter to obtain *Chi1* and *Chi4* chitinase-silenced strains. Hygromycin B resistance gene (HygR) was used to screen transformants. (**B**,**C**) The amplification pattern obtained with primers for the HygR primer sets using genomic DNA isolated from the *C. militaris* transformants and WT. Lane P: plasmid as a positive control; Lane WT: WT strain as a negative control.

To clarify the role of chitinases in fruit body development, *Chi1* and *Chi4* were selected to construct the gene silencing strains. The gene-silenced transformants were screened phenotypically on PDA supplemented with hygromycin B, and the results were confirmed by PCR (Figure 2A). A ~200 bp DNA fragments with hygromycin B primer sets (HygB-F/HygB-R) was amplified from empty plasmid control (SiControl) and chitinase-silenced strains (*Chi1i* and *Chi4i*), but not in the wild-type (WT) strain. The parallel PCR amplification experiment with plasmid DNA (P) as template was set as positive control (Figure 2B,C).

**Figure 3:**
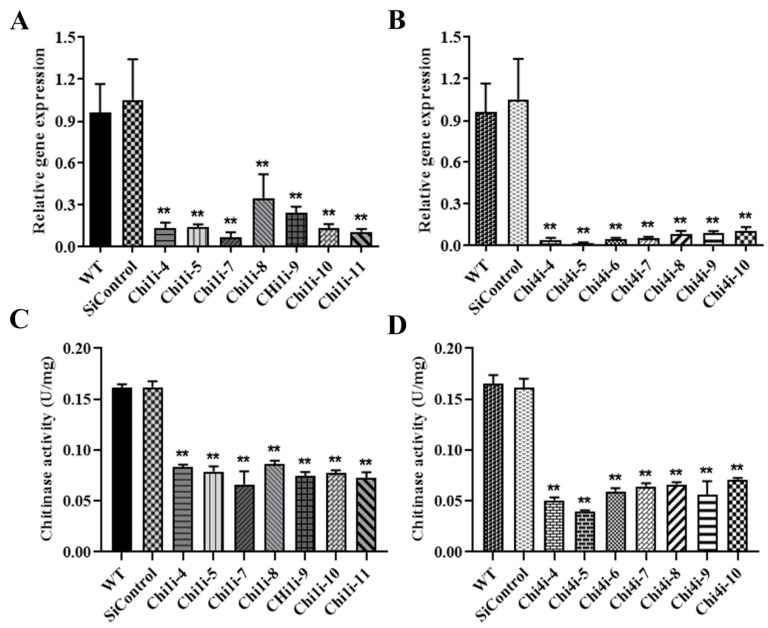
Analysis of enzyme activity and transcription level in *Chi1*- and *Chi4*-silenced strains. (**A**,**B**) qRT-PCR analysis of the relative mRNA levels of the *Chi1* and *Chi4* in *Chi1*- and *Chi4*-silenced strains, respectively. The gene expression of the chitinase was set arbitrarily to one in the WT strain. (**C**,**D**) Determination of chitinase activity of WT and chitinase-silenced strains. Standard errors and means were determined from three independent replicates. Asterisks indicate significant differences compared to the WT strain according to two-way analysis of variance using GraphPad Prism (*n* = 3, ** *p* < 0.01).

The chitinase gene transcription level in *Chi1i* and *Chi4i* strains were significantly decreased approximately 60–80% and 80–90% compared to WT and SiControl strains, respectively (Figure 3A,B). The chitinase activity of *Chi1i* and *Chi4i* strains were significantly decreased approximately 45–60% and 60–70% than WT and SiControl, respectively (Figure 3C,D). The *Chi1i* strains, *Chi1i-4*, *Chi1i-9*, and *Chi4i* strains, *Chi4i-5*, and *Chi4i-6* were randomly selected to investigate the role of chitinase in fruiting body development in *C. militaris*.

### 3.3. Chitinase 1 and 4 Is Involved in Fruit Body Formation of C. militaris

**Figure 4:**
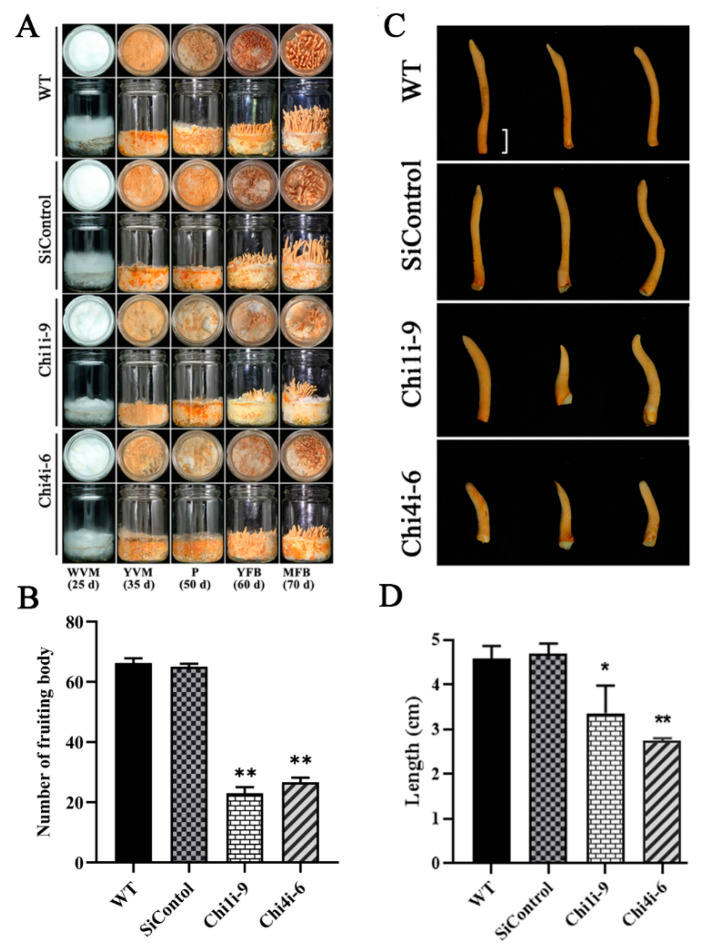
Detection of fruiting body development in *Chi1*- and *Chi4*-silenced strains. (**A**) The fruiting body developmental phenotype in WT, SiContol, *Chi1i*-9 and *Chi4i*-6 strains. (**B**) Number of fruiting body in one bottle of WT, SiContol, *Chi1i*-9 and *Chi4i*-6 strains. Asterisks indicate significant differences compared to the WT strain according to two-way analysis of variance using GraphPad Prism (*n* = 3, ** *p* < 0.01). (**C**,**D**) The morphology and length (bar: 1 cm) of single fruiting body in WT, SiControl, *Chi1i*-9 and *Chi4i*-6 strain. Asterisks indicate significant differences compared to the WT strain according to two-way analysis of variance using GraphPad Prism (*n* = 3, * *p* < 0.05, ** *p* < 0.01).

Next, we used genetic tests to detect the role of chitinase in the fruiting body development of *C. militaris*. The two *Chi1*-silenced and *Chi4*-silenced strains exhibited consistent results. Thus, we present only the results of the WT, SiControl, *Ch1i-9* and *CH4i-6* strains in the following results. As shown in Figure 4A, the mycelium of WT and SiControl strains changed from white to yellow, and then kinked to form the primordium, and further formed the young fruiting body and mature fruiting body. When the chitinases were silenced, the degree of mycelium color from white to yellow is weakened, the differentiation of the primordium was defective, and the number of fruiting body was significantly decreased approximately by 50% (Figure 4B). Compared with WT and SiControl strains, the length of the single mature fruiting body was shortened by 27% and 38% in *Chi1*- and *Chi4*-silenced strains, respectively (Figure 4C,D). The damage of fruiting body formation may be caused by the lack of chitinase that degrades the stratum corneum, leading to the damage of primitive differentiation. These results indicated that chitinase involved in fruiting body development of *C. militaris*.

### 3.4. Increased Cell Wall Chitin Content and Thickness in Chitinase Silenced Strains

**Figure 5:**
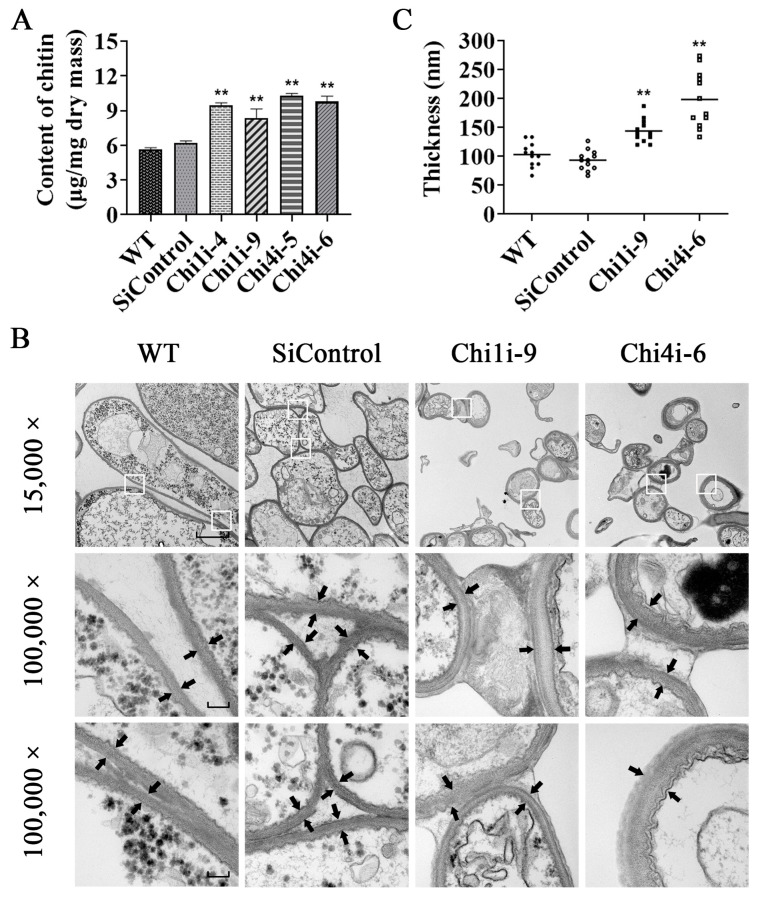
Transmission electron microscopy (TEM) analysis of cell wall thickness in chitinase-silenced strains and WT. (**A**) Determination of the chitin content in chitinase-silenced strains. Standard errors and means were determined from three independent replicates. Asterisks indicate significant differences compared to the WT strain according to two-way analysis of variance using GraphPad Prism (*n* = 3, ** *p* < 0.01). (**B**) Thickness of the cell wall in chitinase-silenced strains. There were three biological replicates for each fungal strain (representative pictures are shown). For each line, at least 30 single cell TEM images were captured. The magnifications are 15,000× (bar: 2 μm) and two fields with white frames are further enlarged (100,000×, bar: 200 nm). The black arrows show the inner and outer edges of the cell wall layers. (**C**) Statistical plot of cell wall thickness quantification. Asterisks indicate significant differences compared to the WT strain according to two-way analysis of variance using GraphPad Prism (*n* = 3, ** *p* < 0.01).

Our results showed that the fruiting body development is defective in chitinase-silenced strains. Because the *C. militaris* cell walls are mainly composed of chitin, we assessed chitin contents in WT, SiControl, and chitinase-silenced strains. Compared to WT and SiControl strains, the contents of chitin in strains *Chi1i*-9 and *Chi4i*-6 were significantly increased approximately by 1.6-fold and 1.8-fold, respectively (Figure 5A). Since the cell wall structure or composition changes to some extent, it can be presented through the ultrastructure of the cell wall. Therefore, the thickness of cell wall was measured by transmission electron microscopy (TEM) to further detect the changes of cell wall after chitinase gene inhibition. Compared with WT strain, the cell wall thickness of *Chi1i*-9 and *Chi4i*-6 was significantly increased approximately by 1.4-fold and 1.9-fold, respectively (Figure 5B,C). These results hint that the increased chitin content and cell wall thickness are the causes of defective fruiting body development in chitinase-silenced strains of *C. militaris.*

## 4. Discussion

Chitinase plays a role in chitin catabolism and cell wall remodeling [27]. Some studies implied that chitinase-mediated cell wall remodeling involved in mushroom fruiting bodies development [28,29]. In *Coprinellus komigratus*, the expression of chitinase 2 in autolyzed mature mushrooms was 20 times or twice higher than that in the primordium or young mushrooms, respectively [30]. In *Aspergillus niger*, the chitin mass in the cell wall was significantly increased in the two chitinases *CfcI* and *CtcB* knockout mutant, which was similar to the change of chitin content of *chi1i* and *chi4i* silent strains in *C. militaris* [31]. In *Coprinopsis cinerea*, two chitinases (*Class V ChiB1* and another putative *Class III chitinase gene*) were expressed mainly in the cap during fruiting body maturation, and the expression of these two chitinases increased with the maturation of the fruiting body [32], which was similar to the up-regulation of *Chi1*, *Chi4*, *Chi6*, and *Chi11* transcription in mature fruiting bodies mature fruiting body of *C. militaris.* In addition, we found that silencing of *Chi1* and *Chi4* genes resulted in blocked primordial differentiation and defective fruiting body development, and the chitin content and cell wall thickness were increased. These studies showed that chitinase plays a role in the fruiting body development of different mushroom.

In addition to cell wall remodeling and fruiting bodies development, fungal chitinase participates in the decomposition of chitin in insect cell wall [33]. The main structural component of insect chitin, N-acetyl-β-D-glucosamine, similar in structure to fungal chitin, which is composed of β-(1-4)-linked N-acetylglucosamine (GlcNAc) subunits [34,35]. LysM domain of fungal chitinase were found to participate in chitin decomposition of insects. In fungi *Isaria fumosorosea*, knockout chitinase gene *Ifchit1* which contain LysM domain led to the average lethal dose (LT50) of killing insect *Plutella xylostella* increased by about three times compared with wild-type strains, indicating that *Ifchit1* mutant’s ability to penetrate or destroy insect cuticle was reduced [36]. In insect pathogenic fungus *B. bassiana*, knockout two chitinases *Blys2* and *Blys5*, which containing conservative LysM domain, significantly prolonged the killing time and inhibited the immune response ability to insect wax moth larvae [14]. Knockout another chitinase *BbSir2* of *B. bassiana*, which containing two LysM domains, decreased the budding spore number by about 10–12% and increased the LT50 by 30% of insect *Galleria mellonella* larvae by 30%, showing that *BbSir2* mutant reduced the virulence of *B. bassiana* [37]. In *C. militaris*, the live insect tussah pupa or grain culture medium which added with insect silkworm pupa powder were used to culture fruiting body [38,39]. In this study, conserved domain analysis showed the chitinase *Chi4* and *Chi5* of *C. militaris* also contain LysM domain. Therefore, chitinase of *C. militaris* has the potential to decompose insect chitin which needs further study.

In summary, 14 putative chitinase genes were characterized and formed preliminary studies on the function of chitinase in *C. militaris*. We found primordial differentiation and fruiting body development were defected, and the chitin content and cell wall thickness were increased in *Chi1* and *Chi4* silenced strain. Our results hint that chitinase is involved in the fruiting body development by regulating chitin content in *C. militaris*.

## Data Availability

All experimental data in this study will be made available upon reasonable request from readers.

## Supplementary Materials

The following supporting information can be downloaded at: https://www.mdpi.com/article/10.3390/life13030764/s1, Table S1: The primers used in this study.
