# Supplementary material for: Chitinase Is Involved in the Fruiting Body Development of Medicinal Fungus Cordyceps militaris
Source: Life (Basel). 2023 Mar 12;13(3):764. doi: 10.3390/life13030764 (PMC10051443; doi:10.3390/life13030764)
Supplement: Supplementary file 1 [file life-13-00764-s001.zip › life-2182713-supplementary.pdf]

Table S1. Real-time PCR and *Chi1*, *Chi4* and *HygR* gene silent fragment primer sequences

Primer Name	Sequence (5’-3’)
Chi1-F/R	CCACCTTTTCCGACATTGCT
	CTTTTGCCTGGGGGTATTCC
Chi2-F/R	ATCGTGGTGGCGAAGAGCA
	TGGTGTGGCTCTGGACGAT
Chi3-F/R	TATTGAAGGACTTGCCGCC
	GCTGCTTGTTGAACTGTGT
Chi4-F/R	TGCAAAGACCTTGCGTGCT
	AAGCGACACTGTTTCGTTGGT
Chi5-F/R	CAACCTGCCCTTTTTCATCA
	GTCATTGGCCTTTCCGCT
Chi6-F/R	GTCCGTCTCCGACACAATC
	GCTCTCATCTCGCCCGTTA
Chi7-F/R	AAAAGTCGCCAATGCTACAGC
	TGCAGTGTTGCTGGTCATTGT
Chi8-F/R	CGATTTGCGTTCCGATTC
	TTGGGAGCGGATTGAGAGTG
Chi9-F/R	TCCAGACCTCCACTAACCGA
	CGGCAGGCATATCAATGTG
Chi10-F/R	ACATCAGGCAAACCTCTACCC
	ACAACGGCATACCAAGGACTA
Chi11-F/R	CGCCAGTCCTATCATTTCG
	CATTGTCGTTTGCCATTCC
Chi12-F/R	TCACCAATCACCACACCTCT
	TATTCGTGTCCTTGCCTTCG
Chi13-F/R	TACGGATACTGGGACGAGC
	CTAAGCAAGGCACGAACAC
Chi14-F/R	ATCCGCAACATTGTCCGCTA
	AAACGCCACCTCCATCTGCT
TUB-F/R	ATGTCGTTCGTCGTGAGG
	AGAGTGGCGTTGTAGGGT

Primer Name	Sequence (5’-3’)
Chi1-F/R	CGCggatccTCGAGGTCGAGCCTCTGA
	ACGCgtcgacGATGCGTACTGCGTATTGG
Chi4-F/R	CGCggatccCCCGTCTTCCACATCAATAG
	ACGCgtcgacTAGGGTGGAGGTTTGCGTA
HygR	TTCAGGCTTTTTCAT
	TTTCTTTGCCCTCGG
